# Anti-nucleocapsid antibody levels following initial and repeat SARS-CoV-2 infections in a cohort of long-term care facility residents in England (VIVALDI)

**DOI:** 10.12688/wellcomeopenres.20750.1

**Published:** 2024-02-19

**Authors:** Oliver Stirrup, Gokhan Tut, Maria Krutikov, David Bone, Tara Lancaster, Borscha Azmi, Igor Monakhov, Paul Moss, Andrew Hayward, Andrew Copas, Laura Shallcross

**Affiliations:** 1 Institute for Global Health, University College London, London, England, UK; 2 Institute of Immunology and Immunotherapy, University of Birmingham, Birmingham, England, UK; 3 Institute of Health Informatics, University College London, London, England, UK; 4 UK Health Security Agency, London, UK; 5 Institute of Epidemiology & Health Care, University College London, London, England, UK; 6 Health Data Research UK, London, England, UK

**Keywords:** SARS-CoV-2, COVID-19, Omicron, immunoglobulin G, nucleocapsid

## Abstract

**Background:**

We have previously demonstrated that older residents of long-term care facilities (LTCF) in the UK show levels of anti-spike antibodies that are comparable to the general population following primary series and booster vaccination for severe acute respiratory syndrome coronavirus 2 (SARS-CoV-2). However, data on the humoral response to other SARS-CoV-2 proteins associated with natural infection are scarce in this vulnerable population.

**Methods:**

We measured quantitative levels of anti-nucleocapsid antibodies in blood samples taken from LTCF residents and staff after initial and repeat SARS-CoV-2 infections, between December 2020 and March 2023. Data on SARS-CoV-2 infection and vaccination were obtained through linkage to national datasets. Linear mixed effects models were used to investigate anti-nucleocapsid antibody levels, using log10 scale, in relation to time from most recent infection. This included evaluation of associations between repeat infection, staff/resident status, age, sex, Omicron infection and vaccination history and peak antibody level and slope of decline with time.

**Results:**

We analysed 405 antibody observations from 220 residents and 396 observations from 215 staff. Repeat infection was associated with 8.5-fold (95%CI 4.9-14.8-fold) higher initial (peak) median anti-nucleocapsid antibody level, with steeper subsequent slope of decline. There were no significant differences in antibody level associated with resident (vs. staff) status or age, but Omicron infection was associated with 3.6-fold (95%CI 2.4–5.4-fold) higher levels. There was stronger evidence of waning of antibody levels over time in a sensitivity analysis in which observations were censored in cases with suspected undetected repeat infection.

**Conclusions:**

We found similar levels of anti-nucleocapsid antibody in residents and staff of LTCFs. Repeat infection and infection with an Omicron strain were associated with higher peak values. There was evidence of waning of anti-nucleocapsid antibody levels over time.

## Plain Language Summary

COVID-19 had a severe impact on care homes in the UK early in the pandemic. However, deaths and disease caused by the SARS-CoV-2 virus have decreased over time following successful introduction of vaccinations and resistance linked to prior infection. There has been a lot of research carried out on the body's immune response to the viral spike protein, which was used to create vaccines against the virus. Less is known about our immune response to other proteins produced by the virus, such as nucleocapsid, which have not been used in current vaccines.

We evaluated antibody levels against the viral nucleocapsid protein in older care home residents following initial and repeat SARS-CoV-2 infection and compared these values to those observed in younger care home staff. This was done through a large established cohort study, in which residents and staff of participating homes could volunteer to provide blood samples for analysis.

We found similar levels of antibody levels among staff and older residents of care homes. These findings are in line with previous studies, in which we have shown that care home residents who survive SARS-CoV-2 infection can develop robust immunity. Higher peak antibody levels were observed following repeat infection in both residents and staff.

## Introduction

COVID-19 had a severe impact on UK long-term care facilities (LTCFs) early in the pandemic ^1^. However, mortality and morbidity associated with SARS-CoV-2 has decreased markedly over time following successful introduction of a vaccination regimen ^2^ and accumulating levels of prior infection ^3^.

We reported previously that older residents of LTCFs showed robust antibody and cellular immune responses to primary vaccination series ^4^ and booster vaccination ^5^, which primes the immune system for recognition of the viral spike protein. However, less is known regarding durability of immune response against other viral proteins, such as nucleocapsid, which are not present in current vaccines and for which immune responses are only induced following natural infection. We previously found steeper decline from peak levels of anti-spike antibody in males vs. females following primary 2-dose vaccination among Oxford-AstraZeneca recipients, so sex-linked differences are also plausible for anti- nucleocapsid antibody response and warrant further investigation.

We evaluated the magnitude and rate of decline of anti-nucleocapsid IgG antibody levels in older LTCF residents following initial and repeat SARS-CoV-2 infection and compared these values to those observed in younger LTCF staff.

## Methods

### Patient and Public Involvement (PPIE)

The overall VIVALDI study design was reviewed by representatives of the National Care Forum, an organisation for not-for-profit care providers ^6^. Throughout the study we formed two study-specific PPIE groups consisting of care home staff and relatives of residents, and have visited five different care homes to discuss our study findings, progress and future plans with staff and residents.

### VIVALDI

VIVALDI is a prospective cohort study investigating SARS-CoV-2, including residents and staff of LTCFs (residential and/or nursing) for older adults in England ^6, 7^. Following national guidelines, residents underwent monthly routine polymerase chain reaction (PCR) testing until end of March 2022, when policy switched to symptomatic and outbreak testing only with greater lateral flow device (LFD) use. A subset of participants consented to blood sampling specifically for the VIVALDI study between June 2020 and March 2023 ^3^, with serological testing for IgG antibodies to nucleocapsid protein (Architect (Abbott, Maidenhead, UK) June-2020 to May 2022, Elecsys (Roche Diagnostics, Rotkreuz, Switzerland) July 2022 to March-2023). Quantitative antibody titers against SARS-CoV-2 nucleocapsid IgG were measured using Meso Scale Diagnostics (MSD) V-PLEX COVID-19 (coronavirus disease 2019) Respiratory Panel 1 (catalogue code K15359U) or Panel 2 (catalogue code K15383U) kits (MSD, Rockville, Maryland) from December 2020 onwards. Assay kits were used in accordance with the manufacturer's instructions.

Recruitment for blood sampling was opportunistic and sampling frames to ensure representation by sex or age were not applied. Sample size calculations have been described for the overall VIVALDI study ^7^, but specific calculations were not conducted for this analysis. As most of the overall population of both LTCF residents and staff are female, it was expected that females would comprise the majority of the sampled cohort.

As previously we retrieved all available PCR and LFD SARS-CoV-2 test results from the national testing programme through the COVID-19 Datastore cloud processing platform. Test results and vaccination and hospitalisation data from national records were linked to study participants using pseudo-identifiers based on National Health Service (NHS) numbers. Demographic data including age, sex, care home, and care home role were available from COVID-19 testing records which were entered by requestor during the test registration process (self or by care home staff member).

Residents and staff of participating LTCFs were included if they: consented to blood sampling for the VIVALDI study, at least one sample had undergone MSD analysis, their samples could be linked to routine surveillance data, and at least one MSD result followed detection of initial or second SARS-CoV-2 infection within 1.5 years of positive PCR or LFD test. The 1.5-year cut-off was used to remove the influence of a handful of observations with longer time elapsed, which may have been more susceptible to undetected repeat infection. An individual’s first positive PCR or LFD test in the surveillance dataset was assumed to be their second SARS-CoV-2 infection if preceded by hospitalisation with confirmed SARS-CoV-2 diagnosis or positive anti-nucleocapsid antibody result on Abbott, Roche or MSD assays. A positive test was considered to define a new SARS-CoV-2 infection if >30 days after any previous positive tests. This cut-off was chosen to allow relatively rapid reinfection with emergence of new variants such as Omicron ^8^.

### Consent

Consent processes were led by senior care staff at each care home with general oversight from a provider-specific project manager. This approach was taken primarily because it was not possible for external researchers to visit care homes during the pandemic, and there was an urgent need for data on seroprevalence to inform public health action. Study information was distributed by care providers to all next of kin of residents and followed up with a phone call to check understanding and discuss specific queries relating to the study. For individuals lacking capacity to consent, personal or nominated consultees were identified from next of kin or care home staff, respectively. Participants or their consultees provided written informed consent for blood sampling for research into COVID-19.

### Ethics approval

The study was approved by the South Central – Hampshire B Research Ethics Committee (20/SC/0238) on 29th May 2020. The legal basis to access data is provided by Health Research Authority Confidentiality Advisory Group approval (ref: 21/CAG/0156, granted March 2022).

### Statistical analysis

Log10-transformed MSD anti-nucleocapsid levels were modelled using linear mixed effects models ^4^. Time was expressed in years from most recent infection on PCR or LFD testing, with random intercept terms for each participant. This approach allows for analysis of all available data within a single statistical model, and can accommodate irregular numbers and timings of measurements and potential repeat infection for each participant. Model intercept terms correspond to estimated peak antibody levels and slope terms correspond to rate of decline over time on log10-scale.

An initial model was fitted with independent effects for differences in intercept associated with repeat (vs. initial) infection, resident (vs. staff) status, male (vs. female) and age (/10 years from 60). More complex random effect structures were then considered: random slopes and intercepts model, and separate correlated random intercept terms for initial and repeat infection. Models were then considered with addition of: difference in intercept associated with previous Omicron infection (assumed from 12-December-2021 ^2^), vaccine doses received prior to most recent positive test (1, 2 or 3+ vs. none) and infection within 2 months of booster vaccination, and difference in slope associated with repeat infection, resident status, age and sex. Model comparisons used likelihood ratio tests.

A sensitivity analysis was conducted in which all antibody results suspected to follow undetected repeat infection were censored. An increase in log10-anti-nucleocapsid MSD of 1.96*sqrt(2*σ ^2^) between successive observations was considered to indicate repeat infection, where σ ^2^ is residual variance of the initial analysis model (based on 97.5 ^th^ centile of difference between two independent normal variates).

## Results

**Figure 1.**
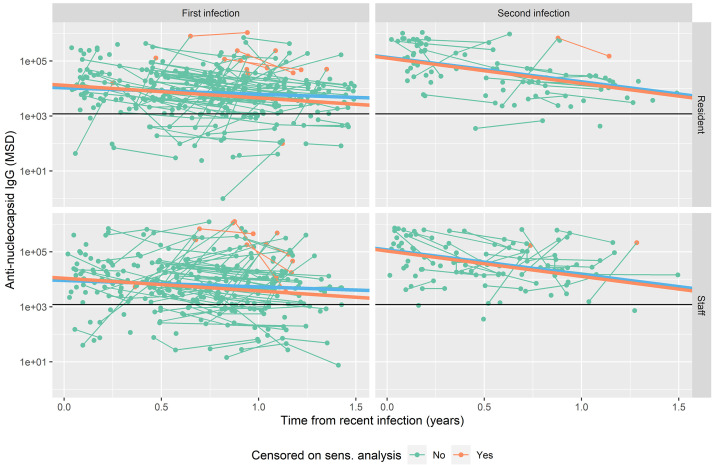
Nucleocapsid-specific antibody levels in relation to time from most recent SARS-CoV-2 infection. Graphs are separated based on first or second infection and staff or resident status. Observations are color-coded by whether they have been censored in the sensitivity analysis because of apparent repeat infection (green: not censored; red: censored). Individual observations are shown as dots, with those from the same person linked by lines. Horizontal black lines show a threshold of 1200 AU/mL to define seropositive status ^4^. Regression lines from a random intercepts model including the categorical variables defining each group are shown for the full dataset (blue lines) and following censoring of suspected repeat infections (orange lines).

Blood samples with MSD measurement of anti-nucleocapsid antibodies were available from 678 residents (471 female, 207 male) and 707 staff (612 female, 95 male) in whom there was linkage to routine surveillance data. Of these 236 residents (167 female, 69 male) and 231 staff (202 female, 29 male) had antibody results following positive SARS-CoV-2 test. Finally, 220 residents (152 female, 68 male) and 215 staff (191 female, 24 male) had at least one antibody result within 18 months of first or second positive test and were included in the analysis ( Figure 1). Matched samples were available from 12 residents (7 female, 5 male) and 12 staff (11 female, 1 male) following both initial infection and a confirmed reinfection.

Residents were majority female (n=152, 69%) with median age of 86.4 (interquartile range (IQR) 77.5–91.5) years (females: 87.0 (80.9–91.9); males: 83.0 (73.2–89.8)). We analysed 312 antibody observations after first and 93 after second infection. Staff were mostly female (n=191, 88.8%) with median age of 49.6 (interquartile range (IQR) 37.6–56.1) years (females: 49.5 (37.5–56.1); males: 51.4 (39.9–55.5)), and within this group we analysed 294 antibody observations after first and 102 after second infection. Primary infection was predominantly with a pre-Omicron SARS-CoV-2 virus in both residents and staff (68% and 57%, respectively), but most second infections were with an Omicron variant (90.8% and 87.9%, respectively).

Regarding development of the mixed effects statistical model for anti-nucleocapsid antibody titre in relation to time from infection, model fit was not significantly improved by random slopes structure (P=0.09) but was improved by separate random intercepts for first and second infection (P=0.009). Omicron infection was found to significantly affect peak antibody level (P<0.001), but no further improvement was seen for vaccine doses received (P=0.19) or infection within 2 months of booster (P=0.58). There was a significant difference in slope of antibody level decline for second *vs* first infection (P<0.001), but no significant effect on this of resident *vs* staff status (P=0.40), age (P=0.82) or sex (P=0.87).

In the final analysis model without censoring ( Table 1) antibody levels declined only slowly following initial infection. Repeat infection was associated with 8.5-fold (95%CI 4.9–14.8-fold) increase in initial (peak) median anti-nucleocapsid antibody level, but steeper subsequent slope of decline such that levels would be similar between first and repeat infections at 1-year after infection ( Table 1). There was no significant difference in antibody level associated with resident status or age, but Omicron infection was associated with 3.6-fold (95%CI 2.4–5.4-fold) higher levels. Applying censoring following suspected undetected infection ( Table 1) revealed similar findings except that antibody levels were seen to decline more rapidly (and with statistical significance) following initial infection.

**Table 1.** Statistical model results for log10-transformed anti-nucleocapsid antibody levels.

	*Initial model*	*Sensitivity analysis with censoring*
	*Model coefficient (95% CI); P*	*Model coefficient (95% CI); P*
Intercept *	3.67 (3.45 to 3.89; <0.01)	3.82 (3.62 to 4.03; <0.01)
Slope (/year) †	-0.09 (-0.28 to 0.10; 0.34)	-0.38 (-0.54 to -0.22; <0.01)
SD(first inf. RE intercept)	0.69 (0.62 to 0.78)	0.81 (0.74 to 0.89)
SD(second inf. RE intercept)	0.41 (0.30 to 0.56)	0.58 (0.49 to 0.70)
Cor(intercept REs)	1.00 (-1 to 1)	0.56 (-0.13 to 0.89)
SD(residual ‘error’)	0.59 (0.55 to 0.63)	0.39 (0.36 to 0.43)
	*Difference in intercept (95%CI) ‡; P*	*Difference in intercept (95%CI) ‡; P*
Second inf. (vs first)	0.93 (0.69 to 1.17; <0.01)	0.82 (0.59 to 1.05; <0.01)
Resident (vs staff)	-0.02 (-0.33 to 0.28; 0.88)	0.06 (-0.26 to 0.38; 0.71)
Male (vs female)	0.07 (-0.12 to 0.25; 0.48)	0.04 (-0.15 to 0.24; 0.67)
Age (/10 yr from 60)	0.03 (-0.04 to 0.10; 0.42)	0.01 (-0.06 to 0.08; 0.81)
Any Omicron inf.	0.56 (0.38 to 0.73; <0.01)	0.42 (0.24 to 0.60; <0.01)
	*Difference in slope (95%CI) ¶; P*	*Difference in slope (95%CI) ¶; P*
Second inf. (vs first)	-0.80 (-1.13 to -0.47; <0.01)	-0.54 (-0.84 to -0.24; <0.01)

Regression coefficients from statistical mixed effects models for log10-transformed Meso Scale Diagnostics (MSD) values for anti-nucleocapsid antibody levels in relation to the time from most recently detected SARS-CoV-2 infection. *Representing average peak value immediately following detection of infection, value for female staff member at 60 years of age following initial infection with pre-Omicron virus. †Value for participant following initial infection. ‡10^x gives multiplicative difference in intercept associated with each factor. **¶**10^x gives multiplicative difference in value at 1 year from peak level.

## Discussion

Optimal immune protection following SARS-CoV-2 infection is likely to require a broad antibody response against a range of viral proteins. Here we studied antibody titres against viral nucleocapsid in staff and residents of LTCFs. Our observations uncover several findings of relevance to potential understanding of the future course of the pandemic. Ageing is associated with immune senescence and as such it was noteworthy that we found similar levels of anti-nucleocapsid IgG antibody levels among staff and older residents of LTCFs. These findings are in line with previous studies in which we have shown that LTCF residents who survive SARS-CoV-2 infection can develop robust immunity, providing protection against subsequent severe outcomes in this vulnerable population, particularly in combination with vaccination ^2^. We did not find evidence of differences associated with sex.

Higher peak antibody levels were also seen following repeat infection, as would be expected for anamnestic immune response. It was somewhat surprising to observe that nucleocapsid-specific antibody responses were higher following Omicron infection compared to infection with a pre-Omicron viral variant. Most Omicron infections were re-infection but this association remained following correction for the enhanced response to second infection and so may indicate a potential association between the biology of omicron viral replication and induction of nucleocapsid-specific immunity. However, it is also possible that the difference could be due to undetected prior infections, which will have been more common for observed Omicron infections. To our knowledge, previous studies have not compared levels according to viral strain.

Comparing quantitative antibody assay results across studies is challenging due to the range of assays in use and lack of standardisation of reporting scales. However, our findings are largely consistent with previous research. Several studies have demonstrated waning of anti-nucleocapsid antibody levels following first detected infection, including the UK Virus Watch cohort study ^9^ and among healthcare workers in the UK ^10^ and Japan ^11^. Scant data are available regarding anti-nucleocapsid antibody levels following repeat infection, but significant boosting in antibody level following repeat infection has been observed in UK healthcare workers ^12^. We do not know of any previous quantitative analyses of anti-nucleocapsid antibody levels among older residents of LTCFs. However, one study of blood donors in the USA with mean age of 52 years found that waning was slower with increasing age ^13^.

There is some evidence that anti-nucleocapsid antibody levels may be lower in people infected after vaccination ^14^, but we did not find an association with vaccine doses received prior to infection. Another analysis found that anti-nucleocapsid antibody levels were lower among people infected within 2 months of booster vaccination (although the subsequent interval to antibody measurement was not reported) ^15^, for which we did not find a significant difference. Previous studies have suggested that anti-nucleocapsid antibody levels peak at 24 days ^10^ or 30–120 ^9^ days after positive test, whereas we modelled a peak level from point of positive test. Evaluation of model residuals did not indicate any substantial problems with model fit, perhaps because we had relatively few observations within a month of positive test ( *n*=30) and participants would not have undergone blood sampling unless fully recovered.

The importance of nucleocapsid-specific antibody titre is of increasing interest. Previous research on the correlation between antibody levels and risk of SARS-CoV-2 infection has focused on the anti-spike antibodies that are also produced in response to current vaccines ^16^. However, there is evidence that the risk of reinfection is negatively associated with anti-nucleocapsid antibody titre in children, indicating a potential direct protective role ^17^. Further research is needed on methods of identifying those still at risk from SARS-CoV-2, despite widespread vaccination and prior exposure to the virus.

The strengths of this study include use of routine SARS-CoV-2 testing data, which included regular asymptomatic PCR testing up to March 2022 and symptomatic and outbreak testing in the following year. This study also included MSD analysis of blood samples obtained over a 2-year period, including repeat observations, from the vulnerable population of older LTCF residents. Limitations of our study include the relatively small number of individuals with repeat infection, with an even smaller number for whom we have antibody observations following both initial and second infection, and a lack of data on comorbidities and ethnicities of participating residents.

Insights into the magnitude and duration of infection-induced immune responses and how this varies by viral strain will help to inform risk assessment for further impact of SARS-CoV-2 among vulnerable populations with high levels of previous exposure to the virus. Our findings suggest that frail older adults who have survived SARS-CoV-2 infection develop robust nucleocapsid-specific antibody responses that are comparable to healthy younger people. Whilst antibody levels subsequently wane, these are boosted strongly by reinfection. It will be of interest to assess their relative importance in prevention of reinfection and/or suppression of clinical severity.

## Data Availability

Raw data cannot be made publicly available, as this was not included within the consent process for participants. The data controllers for this study do not consider any individual-level health data to be fully anonymised, and so intermediary data are not shared beyond the summaries presented in the article. Researchers who would like to access the study dataset, or related stored samples ^6^, can submit an expression of interest on the UCL VIVALDI study website [ https://www.ucl.ac.uk/health-informatics/research/vivaldi/vivaldi-serum-biobank]. Requests will be reviewed by the study team. Ethical approvals must be in place for any further analyses of the samples and data. Any publications must acknowledge study participants and credit the VIVALDI study team. Figshare: STROBE checklist for ‘Anti-nucleocapsid antibody levels following initial and repeat SARS-CoV-2 infections in a cohort of long-term care facility residents in England (VIVALDI)’. https://doi.org/10.5522/04/24828585 ^18^. Data are available under the terms of the Creative Commons Zero "No rights reserved" data waiver (CC0 1.0 Public domain dedication).
