# Supplementary material for: Anti-nucleocapsid antibody levels following initial and repeat SARS-CoV-2 infections in a cohort of long-term care facility residents in England (VIVALDI)
Source: Wellcome Open Res. 2024 Feb 19;9:45. [Version 1] doi: 10.12688/wellcomeopenres.20750.1 (PMC11137476; doi:10.12688/wellcomeopenres.20750.1)
Supplement: Peer review history

# Reviewer report: Reviewer response for version 1

**DOI:** 10.21956/wellcomeopenres.22962.r84354

**Published:** 2024-05-29

**Authors:** Liam Townsend

**Affiliations:** 1 Trinity College Dublin, Dublin, Ireland

This study utilises a subset of data collected as part of the larger VIVALDI study, examining viral and immunological dynamics associated with SARS-CoV-2 infection in LTCF residents and workers. 

 The data collected is mainly related to incident infection, anti-N IgG levels, and very limited demographic data (age and sex are reported). The absence of age having an impact on antibody level is noteworthy.

 While the data analysis is appropriate, the absence of demographics limits this study. It is unclear whether the participants in this sub study are representative of the overall VIVALDI cohort, and as such the generalisability of these results cannot be interpreted. The addition of a comparison table with the cohort as a whole would help address this issue. In particular, the frailty status of participants included in this study would be relevant. Frailty is associated with impaired cellular immunity. The absence of functional assessment of antibodies, e.g., neutralising capacity, should be included in the limitations. Overall, this study highlights the complexity of the immune responses in older individuals to infection post-vaccination, and gives some reassurance regarding quantitative antibody production over time.

Is the work clearly and accurately presented and does it cite the current literature?

Yes

If applicable, is the statistical analysis and its interpretation appropriate?

Partly

Are all the source data underlying the results available to ensure full reproducibility?

Partly

Is the study design appropriate and is the work technically sound?

Yes

Are the conclusions drawn adequately supported by the results?

Partly

Are sufficient details of methods and analysis provided to allow replication by others?

Yes

Reviewer Expertise:

Immunological and phenotype responses to viral infection. Impact of age on the immune response and disease phenotype.

I confirm that I have read this submission and believe that I have an appropriate level of expertise to confirm that it is of an acceptable scientific standard.

# Reviewer report: Reviewer response for version 1

**DOI:** 10.21956/wellcomeopenres.22962.r80457

**Published:** 2024-05-10

**Authors:** Eric J Nilles

**Affiliations:** 1 Brigham and Women’s Hospital, Boston, MA, USA

The study authors report on the humoral immune response to the nucleocapsid protein of SARS-CoV-2 in long-term care facility (LTCF) residents and staff in the UK. The authors explored dynamics of anti-nucleocapsid antibodies (NC) following primary and repeat SARS-CoV-2 infections. Using data from December 2020 to March 2023, the study analyzes NC antibody levels in relation to factors including resident/staff status, age, vaccination profile, and SARS-CoV-2 variant. Key findings suggest that repeat infections lead to higher initial NC antibody levels but with a steeper decline over time, and Omicron infection was associated with higher peak NC antibody levels. Resident vs. staff status and age were not associated with peak NC antibody levels or rate of decline.

 Overall, this is a well written study that adds to the substantial body of evidence around SARS-CoV-2 humoral kinetics. The  methods are clearly laid out, the analyses reasonable, and the discussion and interpretation appropriate. The underlying data and code has not been made available to reproduce the analyses, although the de-identified data may be available upon request. The argument that "de-identified data cannot be made publicly available, as this was not included within the consent process for participants" is not, as far as I'm aware, a requirement in the UK. It would be nice but not essential to consider other modeling approaches to explore non-linear NC kinetics, as has been broadly demonstrated for SARS-CoV-2 anti-spike antibodies, and less so for NC antibodies (e.g. https://wwwnc.cdc.gov/eid/article/28/9/22-0729_article) .

Is the work clearly and accurately presented and does it cite the current literature?

Yes

If applicable, is the statistical analysis and its interpretation appropriate?

Yes

Are all the source data underlying the results available to ensure full reproducibility?

No

Is the study design appropriate and is the work technically sound?

Yes

Are the conclusions drawn adequately supported by the results?

Yes

Are sufficient details of methods and analysis provided to allow replication by others?

Yes

Reviewer Expertise:

infectious disease epidemiology, sero-epidemiology, emerging and re-emerging pathogens.

I confirm that I have read this submission and believe that I have an appropriate level of expertise to confirm that it is of an acceptable scientific standard.

## References

[1] : Longitudinal SARS-CoV-2 Nucleocapsid Antibody Kinetics, Seroreversion, and Implications for Seroepidemiologic Studies. *news-medical.net* .2022;10.3201/eid2809.220729PMC942391735868337
